# Brought with the wind: A case of melioidosis acquired in the United States presenting with multiple pulmonary nodules

**DOI:** 10.1371/journal.pntd.0014790

**Published:** 2026-10-12

**Authors:** Rodrigo Garcia-Santisteban, Maria Kaufman, Hac-Tu Jacqueline Chung, Raphael Khella, Angel Pousa, Winy Kujundzic, Ines Mbaga, Vlad Voin

**Affiliations:** 1 Department of Internal Medicine, Florida Atlantic University, Boca Raton, Florida, United States of America; 2 Miller School of Medicine, University of Miami, Miami, Florida, United States of America; 3 Department of Infectious Diseases, University of Florida, Gainesville, Florida, United States of America; 4 Department of Internal Medicine, Baptist Health South Florida, Boca Raton, Florida, United States of America; Central University of Tamil Nadu, INDIA

## Abstract

*Burkholderia pseudomallei*, the causative agent of melioidosis, is a gram-negative bacterium historically endemic to Southeast Asia and Northern Australia. However, recent environmental detection along the Gulf Coast and an increasing number of reported cases in non-travelers have raised questions regarding potential endemicity and environmental establishment in the Southern United States. We report a case of putatively locally acquired pulmonary melioidosis in a Florida resident with no recent international travel history, illustrating the diagnostic and therapeutic challenges posed by this rare pathogen. The patient presented with fever, pleuritic chest pain, and hypoxia following a period of severe weather. Computed tomography (CT) of the chest revealed multiple solid pulmonary nodules with ground-glass opacities and mediastinal lymphadenopathy. Given his underlying stage 3b chronic kidney disease (CKD), the patient was treated with an intensive course of intravenous ceftazidime followed by oral doxycycline monotherapy, achieving full clinical recovery. Follow-up imaging at 3 months demonstrated complete resolution of the pulmonary nodules. This case emphasizes the importance of considering melioidosis in the differential diagnosis for non-resolving community-acquired pneumonia or significant pulmonary nodules in the Southern United States, even in the absence of international travel. Risk factors such as diabetes mellitus and severe weather events are key clinical clues. While trimethoprim-sulfamethoxazole remains the preferred eradication therapy, doxycycline may serve as a potential alternative for patients with renal impairment when first-line therapy is contraindicated.

## Author summary

We describe a case of melioidosis, a serious infection caused by the bacterium *Burkholderia pseudomallei*, in a Florida resident who had not traveled internationally. Historically, this disease was only found in tropical regions like Southeast Asia and Northern Australia. However, this case adds to a small number of reported infections in individuals without travel history, highlighting the need to remain vigilant for possible local exposures in the Southern United States. Our patient, a 70-year-old man with diabetes and chronic kidney disease, developed severe pneumonia with multiple lung nodules and swollen lymph nodes following a period of heavy rainfall and hurricanes. These weather events can make it easier for individuals to breathe in the bacteria. Because this disease is rare in the United States, it is often misidentified or overlooked, which can be dangerous and lead to delays in treatment. We successfully treated our patient with a long course of antibiotics, including ceftazidime and doxycycline, resulting in complete clinical recovery and total resolution of his lung nodules. This case demonstrates that even when the standard drug, trimethoprim-sulfamethoxazole, cannot be used due to kidney issues, alternative treatments, such as doxycycline, may be considered as potential treatment options. Ultimately, this case serves as a vital reminder for healthcare providers along the Gulf Coast to consider melioidosis in patients with lung infections, even if they have never left the US.

## Introduction

Invasive melioidosis is a severe and frequently underdiagnosed disease caused by the gram-negative environmental saprophyte *Burkholderia pseudomallei (B. pseudomallei)*, a pathogen historically endemic to the tropical climates of Southeast Asia and Northern Australia. Globally, it is estimated to cause approximately 165,000 cases and 89,000 deaths annually [1,2]. Although melioidosis in the United States (US) has historically been associated with international travel, recent domestic cases suggest the pathogen may be establishing an environmental reservoir in the Southern US. Environmental sampling conducted by the Centers for Disease Control and Prevention (CDC) in 2022 identified *B. pseudomallei* in soil and water along the Mississippi Gulf Coast for the first time, suggesting the bacterium may have established environmental persistence within the continental US [3–5]. This report details the eighth documented case of melioidosis in Florida over the past two decades in a patient with no history of recent international travel, highlighting the importance of considering this diagnosis in historically non-endemic regions [6].

## Case presentation

A 70-year-old man with a past medical history of type 2 diabetes, stage 3b chronic kidney disease (CKD), and atrial fibrillation presented to the emergency department with acute, sharp pleuritic chest pain, fever, chills, and a non-productive cough. He denied recent respiratory illness, sick contacts, or international travel. On physical examination, he was febrile (39.9 °C), mildly hypertensive, and hypoxic, requiring 3 liters of supplemental oxygen via nasal cannula. Cardiopulmonary examination revealed decreased breath sounds in the bilateral lower lobes and an irregular heartbeat. Laboratory findings on admission are summarized in Table 1. A viral respiratory panel was negative. Electrocardiogram revealed atrial fibrillation without evidence of acute ischemia. Chest computed tomography (CT) revealed paratracheal and left subcarinal lymphadenopathy alongside multiple solid pulmonary nodules and surrounding ground-glass opacities (Fig 1).

**Table 1:** Admission laboratory findings.

Parameter	Patient Value	Reference Range
White Blood Cell (WBC) count	14.4 K/μl	4.5 – 11.0 K/μl
Neutrophils	86%	40 – 70%
Band Neutrophils	2%	3 – 5%
Erythrocyte Sedimentation Rate (ESR)	49 mm/hr	0 – 15 mm/hr
C-Reactive Protein (CRP)	8.2 mg/dL	< 0.5 mg/dL
Blood Urea Nitrogen (BUN)	46 mg/dL	7 – 18 mg/dL
Serum Creatinine	2.0 mg/dL	0.6 – 1.2 mg/dL

**Fig 1:**
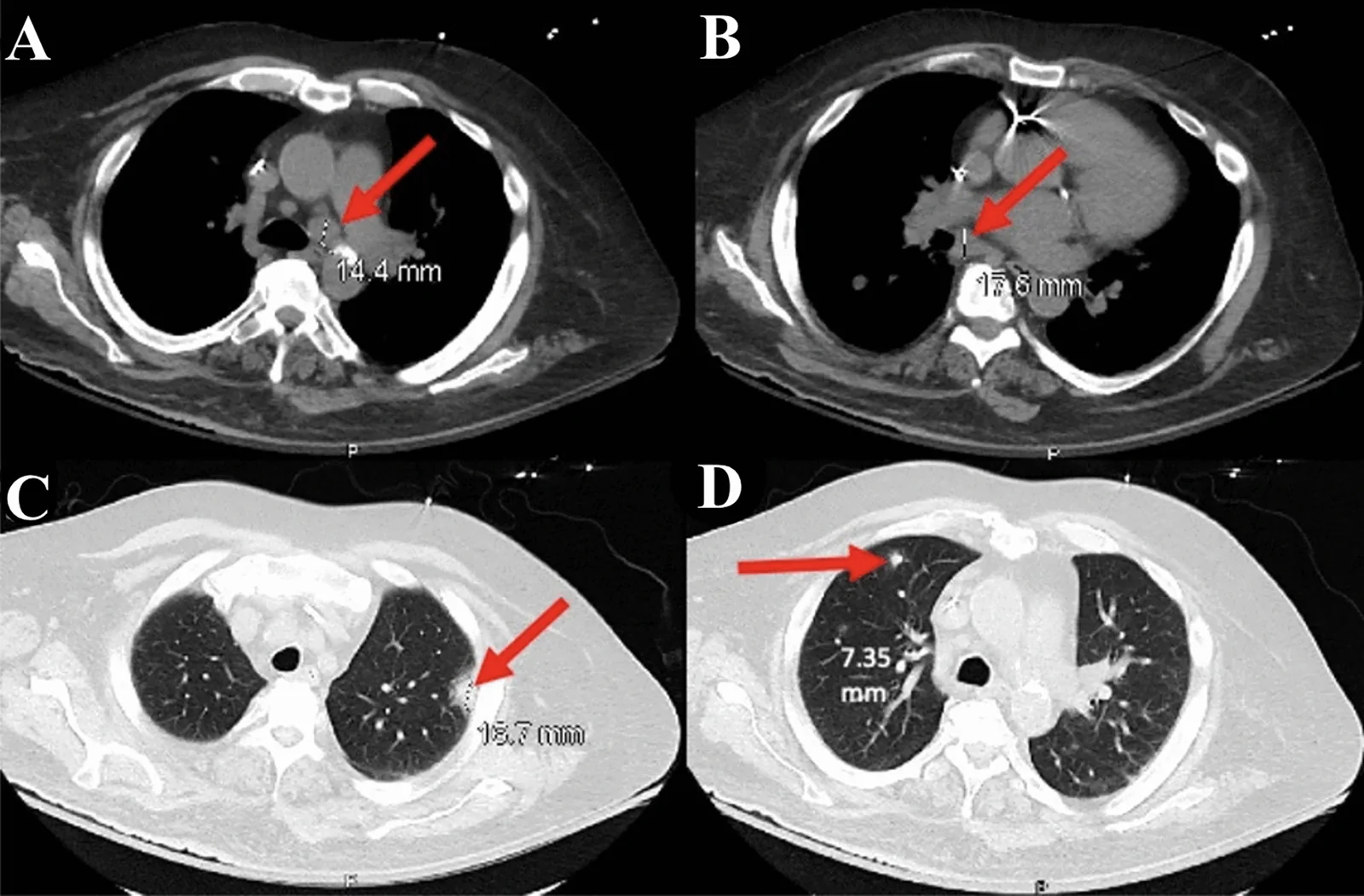
Initial presentation. Chest CT scans demonstrating a paratracheal lymph node measuring 14.4 mm **(A)**, a subcarinal lymph node measuring 17.6 mm **(B)**, a solid pulmonary nodule measuring 16.7 mm **(C)**, and a solid pulmonary nodule measuring 7.35 mm **(D)**.

Empiric antibiotic therapy with intravenous (IV) vancomycin and cefepime was initiated. Initial atypical pathogen tests were negative. At 24 and 48 hours, gram-negative rods were detected in one of two blood cultures and identified as *Burkholderia pseudomallei*. Transthoracic echocardiogram was performed to rule out endocarditis and was negative for vegetations. In accordance with Tier 1 Select Agent laboratory protocols, the blood culture isolate was sent to the Florida Department of Health for definitive confirmation, which verified positive *B. pseudomallei* DNA via nucleic acid amplification (NAA) testing.

Following initial hospital identification of *B. pseudomallei*, antibiotic treatment was adjusted in accordance with European Committee on Antimicrobial Susceptibility Testing (EUCAST) guidelines and CDC management recommendations to high-dose IV ceftazidime (1 gram every 8 hours, adjusted for baseline renal function). The patient exhibited significant clinical improvement. After 4 days of targeted therapy, he was afebrile and maintaining adequate oxygen saturation on room air. He was discharged on IV ceftazidime. Repeat CT imaging at 3-week follow-up showed significant improvement in ground-glass opacities and pulmonary nodules with stable mediastinal lymphadenopathy (Fig 2). After completing a total of 6 weeks of IV ceftazidime, therapy was transitioned to oral doxycycline (100 mg twice daily) for 3 months, selected due to his stage 3b CKD. After 3 months of follow-up, symptoms had resolved completely and repeat CT imaging revealed resolution of the pulmonary nodules and stable mediastinal lymphadenopathy (Fig 3).

**Fig 2:**
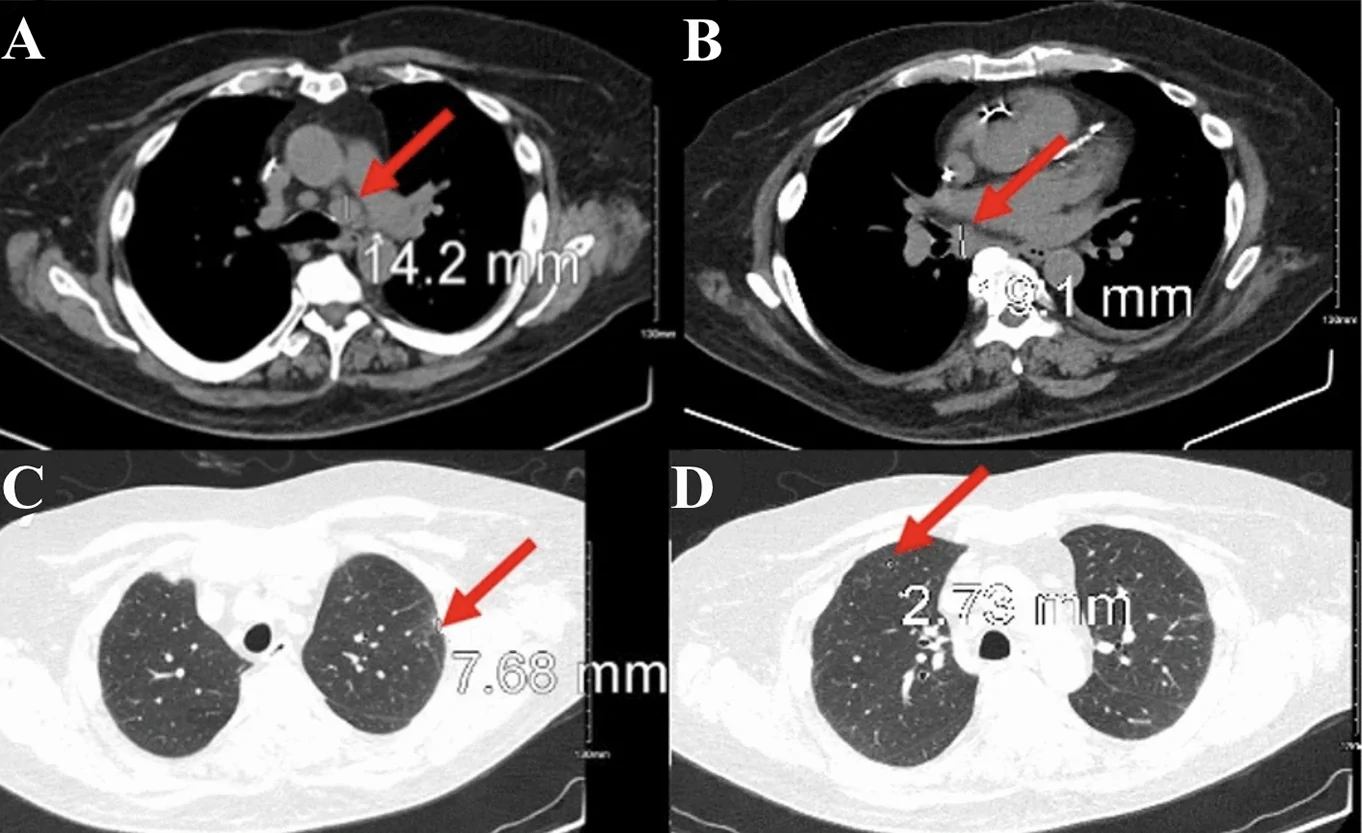
3-week follow-up. Chest CT scans demonstrating stable paratracheal (A) and subcarinal lymph nodes (B) and improved solid pulmonary nodules measuring 7.68 mm from 16.7 mm **(C)**, and 2.73 mm from 7.35 mm **(D)**.

**Fig 3:**
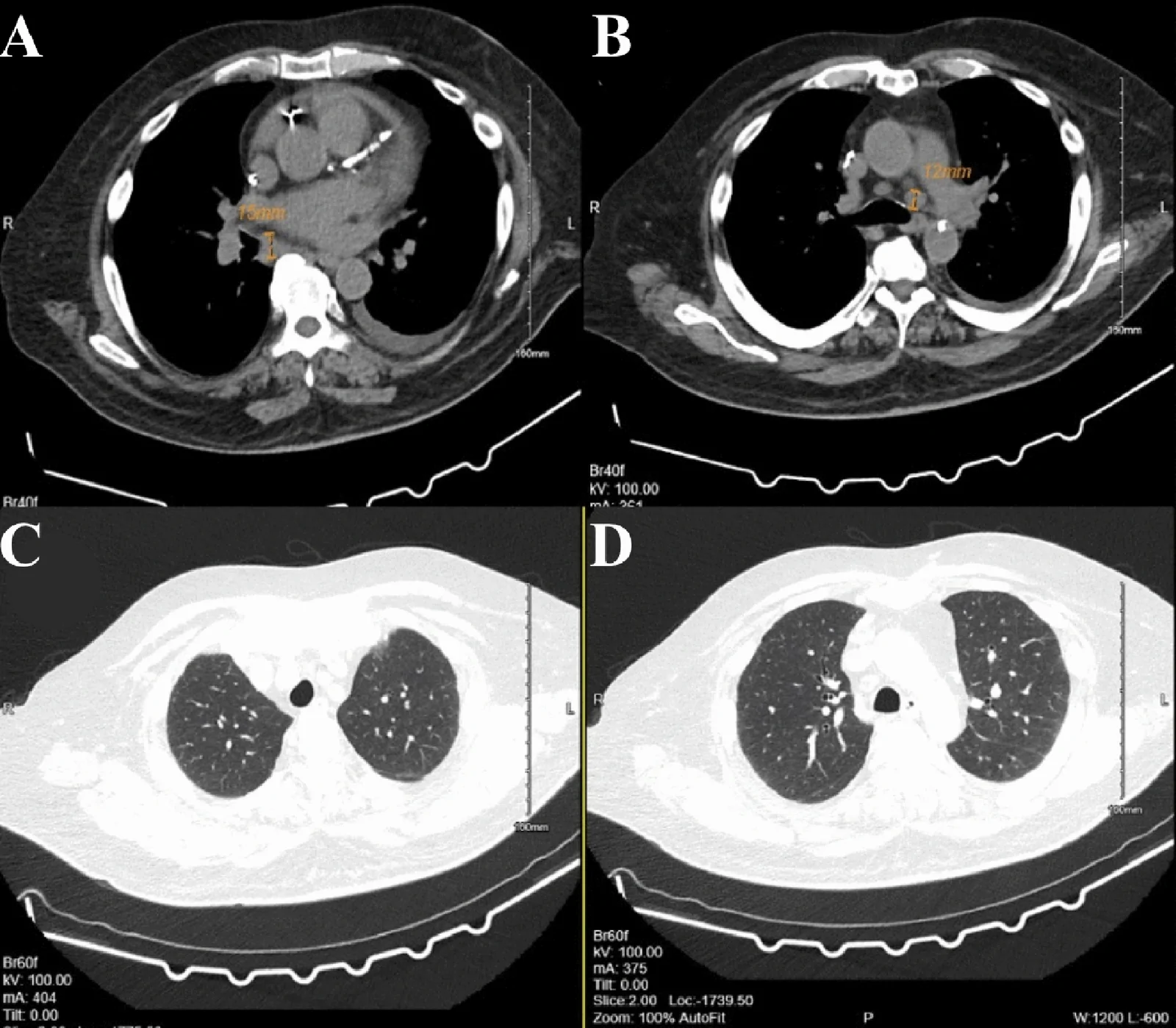
3-month follow-up. Chest CT scans demonstrating stable paratracheal (A) and subcarinal lymph nodes (B) and no signs of previously seen solid pulmonary nodules **(C and D)**.

## Discussion

Melioidosis typically arises from direct contact with, ingestion of, or inhalation of *B. pseudomallei*, a bacterium predominantly reported in Southeast Asia, Australia, and parts of Central and South America [2]. Environmental transmission occurs primarily through exposure to contaminated soil or water, whereas human-to-human and animal-to-human transmission are exceptionally rare [7]. Incubation typically ranges from 1-21 days following exposure; however, in approximately 5% of cases, melioidosis presents from reactivation of a latent infection months to years later [3]. In the US, approximately a dozen cases are documented annually, primarily among travelers to endemic regions [2]. However, there has been rising incidence of locally acquired cases across the US, particularly in the Gulf Coast region. Between 2003 (when melioidosis became a notifiable disease) and 2024, only eight total cases were reported in Florida, with five reported between 2021 and 2024 [6]. While this indicates an increase in documented diagnoses, individual travel-negative cases and localized environmental isolations in neighboring Gulf Coast states cannot alone confirm endemic transmission or environmental establishment within Florida.

In 2022, the CDC identified *B. pseudomallei* in soil and water samples along the Gulf Coast of Mississippi for the first time, suggesting an emerging environmental presence in select regions of the continental US [3]. This discovery followed the investigation of two unrelated, locally acquired cases in southern Mississippi residents without recent international travel [3,4]. Subsequently, another unrelated case was identified in the same geographic region [4,5]. While most US cases remain travel-related [8,9], isolated reports have documented cases occurring without international travel history [10–14], though establishing definitive environmental reservoirs across the Southern US requires dedicated surveillance.

Melioidosis exhibits a broad spectrum of clinical presentations that can range from a nonspecific febrile illness or localized cutaneous abscesses to severe pneumonia with mediastinal lymphadenopathy, endocarditis, meningitis, or fulminant septicemia [15–17]. Known risk factors for severe or invasive disease include diabetes mellitus, chronic kidney disease, excessive alcohol use, chronic lung disease, and other immunosuppressive conditions [16,18]. In our patient, both diabetes and stage 3b CKD may have predisposed the patient and elevated his susceptibility to invasive disease. In fact, diabetes is recognized as the single strongest risk factor and is associated with a 100-fold increased risk of invasive melioidosis [17]. Furthermore, the timing of his presentation following heavy rainfall and hurricane activity in Florida is highly relevant. Intense weather events aerosolize bacteria from the soil and surface water, facilitating deep inhalation and leading to severe pneumonia with pulmonary nodules and mediastinal lymphadenopathy as seen in this case [19,20].

Differential diagnoses for multiple pulmonary nodules with lymphadenopathy are broad and include thoracic malignancies, tuberculosis, fungal infections, or inflammatory granulomatous conditions, such as sarcoidosis and granulomatosis with polyangiitis [21]. Diagnosis is frequently delayed because automated laboratory systems often misidentify *B. pseudomallei* as *B. cepacia* or contaminants. Utilizing advanced molecular diagnostics, including NAA, and state public health reference laboratory testing are essential for rapid, reliable identification of *B. pseudomallei.* Therefore, clinicians in the Southern US must be vigilant in considering melioidosis in patients presenting with non-resolving pneumonia and pulmonary nodules, as early diagnosis and targeted antibiotic treatment are critical for favorable outcomes [1,21].

Management of *B. pseudomallei* infection follows a standardized two-phase approach divided into an intensive phase followed by an eradication phase, guided by CDC and EUCAST guidelines [22,23]. The intensive phase requires a minimum of 2 weeks of IV ceftazidime or meropenem [21,24]. In our patient, the extension of the IV phase to 6 weeks was clinically indicated due to the burden of pulmonary nodules and the patient’s comorbid risk factors. The subsequent eradication phase requires 3–6 months of oral trimethoprim-sulfamethoxazole (TMP-SMX) or doxycycline [21,24]. While large-scale trials have demonstrated that TMP-SMX monotherapy is significantly more effective than doxycycline monotherapy in preventing relapse, doxycycline was selected due to the patient’s stage 3b CKD, which put him at risk of hyperkalemia and renal function deterioration with TMP-SMX use [25]. This strategy is supported by recent data from 2023 indicating that nearly 25% of patients treated with TMP-SMX for melioidosis experience significant adverse events requiring a drug switch, emphasizing the importance of tailored therapy [26]. The patient’s full clinical and radiological recovery highlights doxycycline monotherapy as a potential alternative when first-line agents are contraindicated [25], though broader conclusions regarding generalizable efficacy cannot be draft from an individual case. Strict adherence during the eradication phase must be emphasized with proper patient counseling on compliance because premature termination carries a high risk of disease relapse [1,21]. For individuals with high-risk environmental exposures, postexposure prophylaxis with oral TMP-SMX or amoxicillin/clavulanic acid for 21 days is recommended [27].

Confirmed cases of melioidosis must be reported immediately to state public health authorities and the CDC [28]. *B. pseudomallei* is classified as a Tier 1 Select Agent and Category B bioterrorism agent due to its potential for misuse and severe public health impact [7,28]. Given the challenge of identifying environmental sources of *B. pseudomallei* from established soil and water, clinical focus must shift towards awareness, diagnostic detection, and early therapeutic intervention in non-endemic areas [3].

This report has limitations inherent to single-case studies, preventing direct generalizability to all patient populations. Furthermore, local environmental sampling of soil and water near the patient’s residence was not performed to definitively isolate the environmental source. Finally, while the patient achieved complete clinical and radiological recovery at 3 months, long-term surveillance for potential late relapses remains ongoing.

## Conclusion

This case underscores the shifting epidemiology of melioidosis in the US. As *B. pseudomallei* continues to demonstrate an environmental presence in the Southern US soil, melioidosis can no longer be viewed strictly as a “traveler’s disease”. While a single case cannot confirm ongoing environmental establishment in Florida, healthcare providers in the Gulf Coast region must include melioidosis in the differential diagnosis for community-acquired pneumonia featuring pulmonary nodules or mediastinal lymphadenopathy, especially in patients with predisposing comorbidities like diabetes or CKD or environmental exposure following severe weather events. Finally, while TMP-SMX remains the established gold standard for the eradication phase of treatment, this case demonstrates that oral doxycycline represents a potential alternative in the setting of significant renal impairment when TMP-SMX is contraindicated.
